# Investigation of EZH2 pathways for novel epigenetic treatment strategies in oropharyngeal cancer

**DOI:** 10.1186/s40463-016-0168-9

**Published:** 2016-10-28

**Authors:** Sherif Idris, Cameron Lindsay, Morris Kostiuk, Colin Andrews, David W. J. Côté, Daniel A. O’Connell, Jeffrey Harris, Hadi Seikaly, Vincent L. Biron

**Affiliations:** 1 Division of Otolaryngology-Head & Neck Surgery, Department of Surgery, University of Alberta, 1E4 Walter Mackenzie Centre, 8440-112 Street NW, Edmonton, AB Canada T6G 2B7; 2 Alberta Head & Neck Centre for Oncology and Reconstruction, 1E4 Walter Mackenzie Centre, 8440-112 Street NW, Edmonton, AB Canada T6G 2B7

**Keywords:** Epigenetics, EZH2, Histone methylation, Oropharyngeal cancer, Squamous cell carcinoma, GSK-343, 3-deazaneplanocin A, EPZ005687

## Abstract

**Background:**

In recent decades, the incidence of oropharyngeal squamous cell carcinoma (OPSCC) has been rising worldwide as a result of increasing oncogenic human papillomavirus (HPV) infections in the oropharynx. EZH2 is an epigenetic regulatory protein associated with tumor aggressiveness and negative survival outcomes in several human cancers. We aimed to determine the role of EZH2 as a potential therapeutic epigenetic target in HPV-positive and negative OPSCC.

**Methods:**

The expression of EZH2 was measured by immunohistochemistry (IHC) and droplet digital PCR (ddPCR) in 2 HPV-positive and 2 HPV-negative cell lines. The cell lines were then cultured and treated with one of 3 EZH2 epigenetic inhibitors (3-deazaneplanocin A, GSK-343 and EPZ005687) or DMSO (control). Following 2, 4 and 7 days of treatment, cells were analyzed and compared by gene expression, cell survival and proliferation assays.

**Results:**

EZH2 targeting resulted in greater inhibition of growth and survival in HPV-positive compared to HPV-negative cells lines. The expression profile of genes important in OPSCC also differed according to HPV-positivity for Ki67, CCND1, MET and PTEN/PIK3CA, but remained unchanged for EGFR, CDKN2A and p53.

**Conclusion:**

Inhibition of EZH2 has anti-tumorigenic effects on OPSCC cells in culture that is more pronounced in HPV-positive cell lines. EZH2 is a promising epigenetic target for the treatment of OPSCC.

## Background

Epigenetic deregulation of cellular programs is a key hallmark of human cancers [1–4]. Epigenetics relates to any process that alters gene activity without changing the deoxyribonucleic acid (DNA) sequence, and leads to modifications that can be transmitted to daughter cells. These modifications include DNA-methylation, non-coding RNAs and a variety of histone post-translational modifications [5]. Of the later category, histone methylation has been of particular interest for cancer research. Histone methylation provides stable markers of gene expression, turning genes “on” or “off” for the maintenance of specific cell lineages [6]. Deregulation of histone methylation results in altered tumorigenic gene expression profiles that can be reproducibly identified for diagnostic purposes and specifically reversed for novel cancer therapeutics [6–8]. Recent advances in cancer epigenetics are showing promising implications for the development of personalized oncologic care [9]. In head and neck squamous cell carcinoma, the importance of epigenetic cellular modifications has only recently become apparent.

In recent decades, the incidence of oropharyngeal squamous cell carcinoma (OPSCC) has been increasing worldwide as a result of oncogenic human papillomavirus (HPV) [10, 11]. HPV-positive and HPV-negative OPSCCs are distinct from clinical, pathological and molecular perspectives [12–19]. HPV-positive tumors overexpress p16 (surrogate marker of HPV–positive OPSCC), now used clinically as a significant predictor of improved survival [13]. Recent studies including our own work, has shown significant epigenetic differences between HPV-positive and HPV-negative OPSCC [20, 21]. Histone methylation has been of particular interest, given the importance of these modifications in other cancers. Histone H4 lysine 20 trimethylation (H4K20me3) has been shown to be lost as a hallmark of human cancer [2], and is differentially decreased in p16-positive OPSCC [20] The enzyme enhancer of zeste homolog 2 (EZH2) is a histone methyltransferase (HMT) that acts to trimethylate lysine 27 on histone H3 (H3K27me3) and its increased expression has been linked to a number of malignancies. EZH2 has been extensively studied in several cancers and has been shown to play a significant role in the development of breast cancer, lymphoma, gastric cancers, esophageal cancer, non small-cell lung carcinomas, soft tissue sarcomas, salivary gland adenoid cystic carcinoma, and head and neck carcinomas. In oral cavity cancers (OCSCC), elevated EZH2 expression correlates with more clinically aggressive tumors that result in poorer survival outcomes [22]. In OPSCC, which are molecularly and clinically distinct from OCSCC the role of EZH2 in remains unclear, but may involve an interaction with the HPV-E7 oncoprotein [23, 24].

Overexpression of EZH2 is correlated with advanced disease and a poor prognosis in HNSCC [22]. This is believed to be secondary to an inhibitory effect of EZH2 on the expression of tumor suppressor genes [25]. Therefore, EZH2 inhibition has been regarded as an attractive drug target through the reactivation of tumor suppressor genes We therefore aimed to investigate the role of this epigenetic regulator as a potential therapeutic target in OPSCC. To this end, three EZH2 inhibitors (GSK-343, EPZ005687 and DZNeP) were investigated for their inhibitory effects on EZH2 levels in HPV-positive and HPV-negative HNSCC cell lines. To date, the use of these inhibitors has not been reported in OPSCC.

## Methods

### Oropharyngeal carcinoma tissue

Fresh OPSCC tissues were obtained from patients undergoing primary surgical treatment, as per an approved University of Alberta health ethics research protocol (Pro00016426). Tissues were collected and stored as previously described [26].

### Cell culture and drug treatment protocol

SCC-9 and SCC-104 cell lines were cultured using recommended conditions [24]. Cells were sub-cultured using common techniques with 0.25 % Trypsin/EDTA.

Cells subjected to drug treatments were seeded at ~ 25 % confluency and allowed to settle and recover for 3 days. On the third day the cells were treated with fresh media containing the various inhibitors, DMSO alone or media with no additives. Cells were treated for 7 days, with fresh media/drugs added on the third day and harvest occurring on day 7.

### Histone enrichment and western analysis

Histones were enriched from cell lysates following the AbCam protocol “Histone extraction protocol for western blot” (reference http://www.abcam.com/protocols/histone-extraction-protocol-for-western-blot).

Following incubation the tubes were briefly vortexed, then centrifuged for 10 min at 6500 x g and 4 ° C. The histone containing supernatant (~400 ul) was transferred to new tubes and 100 ul of Trichloroacetic acid was added. The tubes were vortexed briefly and incubated on ice for 1.5 h followed by centrifugation for 15 min at 16,000 x g and 4 ° C. The supernatant was aspirated and the pelleted protein was washed twice with -20 °C acetone and allowed to dry with the lid open at room temperature for 1.5 h. Pelleted histones were either stored at -20 ° C or immediately subject to SDS-PAGE and Western blot. A target of 5 μg of protein was used for Western blotting. Membranes were incubated for 1 h at ambient temperature with a 1:1000 dilution of anti-H3K27me3 primary antibody (monoclonal mouse) in PBST + 3 % milk solution. Membranes were washed extensively in PBST and incubated in 1:5000 HRP-conjugated anti-mouse secondary antibody (polyclonal goat) for 1 h at room temperature in PBST + 5 % milk. Membranes were washed extensively with PBST and treated with Amersham ECL Western Blotting Detection Reagents kit (cat:28-392766, GE Healthcare).

### RNA extraction, purification and ddPCR

RNA purification was performed using the RNeasy Plus Mini Kit (Qiagen). The 20 μl of sample was homogenized using the QIAshredder (Qiagen). 40 ng of RNA was used to synthesize cDNA using the iScriptTM Reverse Transcription Supermix for RT-qPCR (BIO-RAD) as per the manufacturer’s protocol. Following the reaction the cDNA was diluted with Nuclease-free H20 to 1 ng/μl and either stored at -20 °C or used directly for ddPCR as described previously [26]. Primers/probes for the detection of EGFR, MKi67, MET, TP53, CCND1, PTEN, PIK3C and EEF2 were obtained from BIO-RAD, using their recommended protocol. Reactions were set up in a 96 well plate according to the QX200 Droplet Generator Instruction Manual (#10031907 BIO-RAD).

### Immunofluorescence

Cell lines were cultured and treated as per “Drug Treatment Protocol” in 6-well cell culture plates containing glass microscope cover slips coated with poly-L-lysine. A standard immunohistochemistry protocol was followed as recommended by AbCam using the following antibody concentrations. SCC-9 primary antibodies contained 1:100 dilutions of anti-EZH2 and 1:400 dilutions of anti-H3K27me3. SCC-104 primary antibodies contained 1:50 dilutions of anti-EZH2 and 1:400 dilutions of anti-H3K27me3 SCC-9 secondary antibodies contained 1:400 dilutions of anti-Rabbit-Alexa 647 and 1:400 dilutions of anti-Mouse-Alexa 488 . SCC-104 secondary antibodies contained 1:1000 dilutions of anti-Rabbit-Alexa 647 and 1:400 dilutions of anti-Mouse-Alexa 488 Coverslips were mounted in ProLong Gold antifade reagent containing DAPI (4’,6-diamidino-2-phenylindole; Invitrogen) and imaged on an Aperio FL Scancope FL Images (12-bit) were analyzed using Aperio ImageScope v12.2 and HALO v1.95 with manufacturer’s algorithms..

## Results

### EZH2 expression in OPSCC patient tumors

**Fig. 1:**
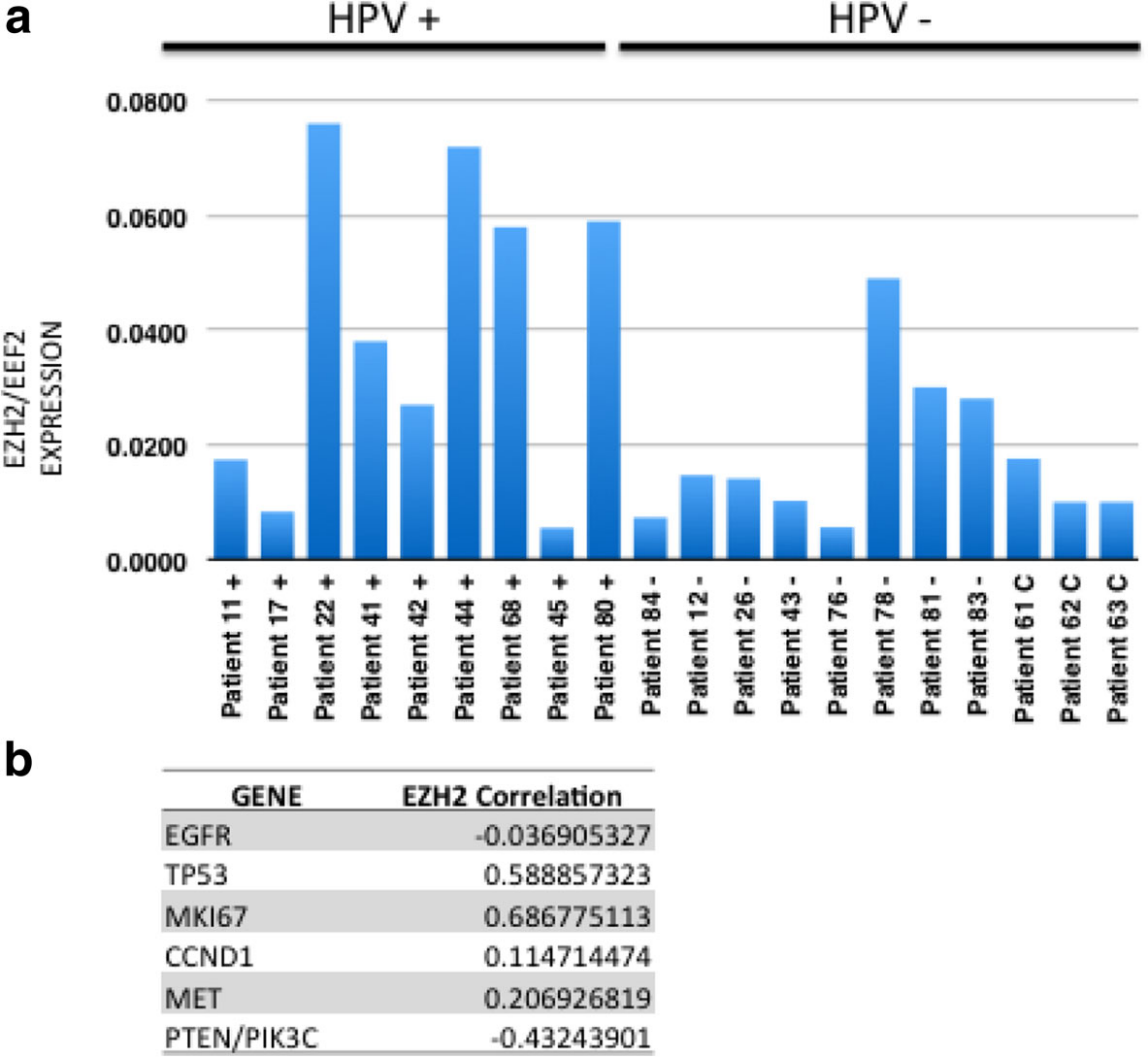
EZH2 expression in HPV+ and HPV- oropharyngeal cancer patients. **a** EZH2 RNA expression from tissue biopsies in HPV+ and HPV- oropharyngeal patient tissues measured by ddPCR absolute levels relative to EEF2 (EZH2:EEF2 ratio). Significant differences are seen between HPV+ and HPV- patients (*p* = 0.006). HPV positivity was determined by clinical p16 immunohistochemistry and confirmed by HPV-16 E6 and E7 ddPCR. **b** Pearson correlation of EZH2 expression in each patient tissue above with EGFR, TP53, MKI67, CCND1, MET and PTEN/PIK3C

As an initial step to investigate the potential role of EZH2 in OPSCC, we compared the RNA expression of EZH2 in a panel of randomly selected HPV+ and HPV- patient tumor samples (Fig. 1). The EEF2 standardized expression of EZH2 was found to vary significantly between patients. The mean expression level of *EZH2* was significantly higher in HPV+ vs HPV- tumors (*p* = 0.006). However, within both groups, there was a broad range of *EZH2* RNA levels. Levels of EGFR, TP53, MKi67, CCND1, MET and PTEN/PIK3C were correlated to EZH2 levels in these patient tissues. Significant positive correlations were seen between elevated EZH2 levels and TP53 (*r* = 0.58) and MKi67 (*r* = 0.69).

### Inhibition of EZH2 in HNSCC cell lines

**Fig. 2:**
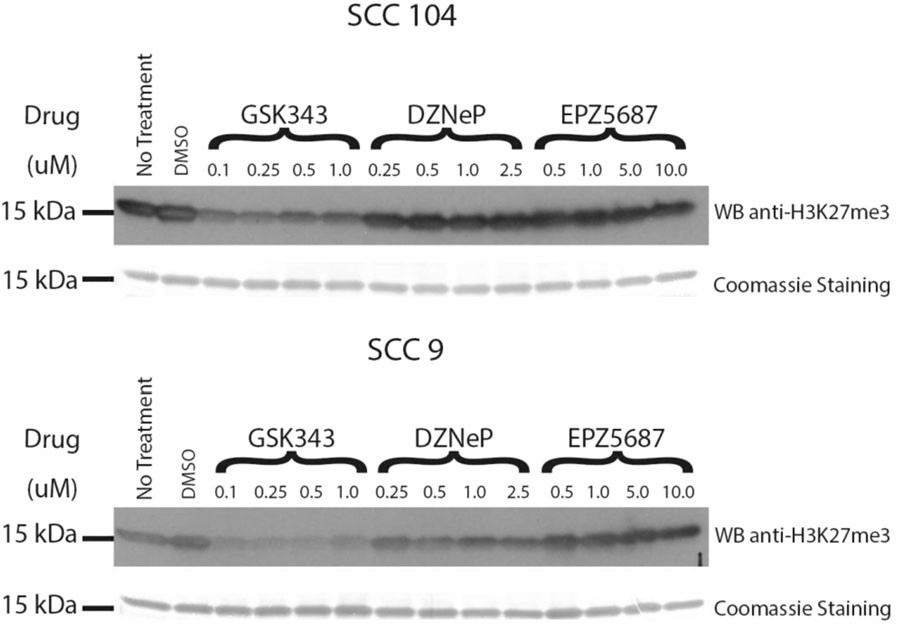
H3K27me3 levels in HPV positive and negative HNSCC cell lines treated with EZH2 inhibitors. Western blot of H3K27me3 levels from of histone extrations (TCA precipitated) in SCC 104 cells (*top*) and SCC 9 cells (*bottom*) following 7 days of growth post-treatment with increasing concentrations of drug as shown. Coomasie stain is shown below each blot demonstrating equal protein loading

In HNSCC cell lines, significant reductions in H3K27me3 (EZH2 substrate) were seen on western blots in response to epigenetic inhibitors, with differences seen between HPV+ and HPV- cells (Fig. 2). However, no appreciable reductions in H3K27me3 could be seen with EPZ5687, comparable to DMSO or untreated cells. In SCC104 cells, GSK343 treatment caused the greatest relative reduction in H3K27me3, with no measurable changes resulting from DZNeP or EPZ5687, even at drug concentrations >2x higher than recommended by the manufacturer. In SCC9 cells, GSK343 treatment also caused a similar reduction in H3K27me3 levels, with a slight reduction from DZNeP.

**Fig. 3:**
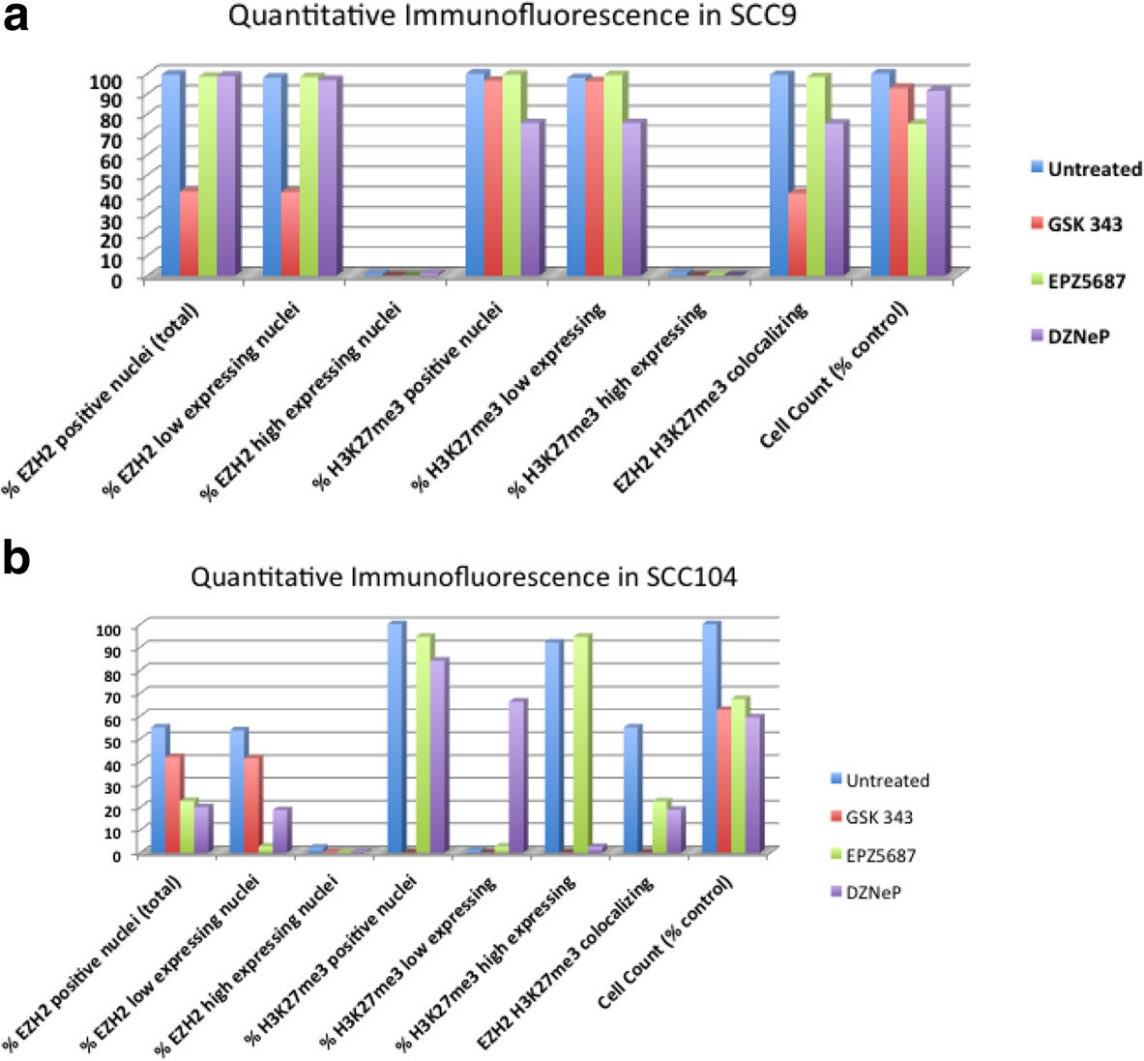
Immunofluorescence analysis of HPV positive and negative cells treated with EZH2 inhibitors. **a** SCC9 and **b** SCC104 cells processed for immunofluorescence with DAPI, H3K27me3 and EZH2 7 days post treatment with GSK 343 (0.5 uM), EPZ5687 (5uM) or DZNeP (1uM) or DMSO (untreated) were imaged and quantified using HALO. Cell counts are shown relative to untreated cells at day 7

We examined the expression of EZH2 and H3K27me3 in SCC9 and SCC104 cells in response to epigenetic inhibitors by immunofluorescence microscopy and quantitative imaging analysis. In SCC9 cells, GSK343 resulted in a >58.3 % reduction in EZH2 positive nuclei (compared to control intensity) with a slight reduction in H3K27me3 levels (3.4 %) (Fig. 3a). EPZ 5687 did not appear to have a notable impact on EZH2 or H3K27me3 levels. DZNeP treated SCC9 cells had a minimal reduction in EZH2 positive nuclei with a ~ 25 % reduction in H3K27me3. In SCC104 cells, GSK343 reduced the amount of EZH2 positive nuclei by ~ 20 % while cause a near complete reduction in H3K27me3 levels (Fig. 3a). Although EPZ5687 and DZNeP showed a greater reduction in EZH2 positive nuclei, this resulted in a minimal reduction in H3K27me3.

Fluorescence quantification of nuclei enabled for a comparison of cell counts between treated and untreated cells, providing an estimate of cell death. In SCC9 cells, GSK 343 and DZNeP treated cells had ~ 10 % reduction in cell counts, whereas EPZ5687 treated cells had ~ 25 % reduction (Fig. 3a). In SCC104 cells, all three inhibitors reduced cell counts by ~ 40 % (Fig. 3b).

## Discussion

This is the first study to investigate EZH2 inhibition as a potential epigenetic chemotherapy for OPSCC. As with OCSCC [25], we have shown that the expression of EZH2 is variable between patients with OPSCC but more often elevated in HPV + OPSCC. With the rising incidence of HPV+ OPSCC [27], the identification of novel chemotherapeutics for this disease is becoming ever more important. Our comparison or three EZH2 inhibitors as such potential novel therapeutic agents has identified GSK-343 as an effective inhibitor of EZH2 and its substrate H3K27me3 in both HPV+ and HPV- cell lines. GSK-343 has also been recently reported to be an effective growth suppressor in HPV+ cervical cancer cell lines. Importantly, this competitive inhibitor of EZH2 has been shown to highly selective at exceedingly low concentrations, [28] with a significantly higher affinity for EZH2 than EPZ5687 [29, 30]. GSK-343 is therefore a promising novel therapeutic drug warranting further investigation for use in HPV + OPSCC.

Epigenetic aberrancies involving histone methylation are a hallmark of human cancers [2, 5, 31–34]. Histone methyltransferases, the enzymes that add methylation to histones, are central regulators of gene expression that are often altered in human malignancies. Because of their specific catalytic activity that can be targeted, these enzymes have been of tremendous interest for the development of novel chemotherapies [7, 33–38]. The histone methyltransferase EZH2 catalyzes H3K27 trimethylation, leading to transcriptional inactivation of target genes [22]. Overexpression of EZH2 has been shown to have an important role in various malignancies, including breast, prostate, gastric, hepatic, and bladder carcinoma [16]. Studies examining the role of EZH2 overexpression in HNSCC have been largely limited to OCSCC. Zhong et.al, demonstrated a statistically significant correlation between the overexpression of EZH2 and tumor proliferation, pathologic grade and nodal metastases in patients with oral tongue cancer. EZH2 has also been shown serve as an independent prognostic marker for overall survival in patients with OCSCC, with high EZH2 expression being significantly associated with poorer survival outcomes [22]. In OPSCC, which is molecularly and clinically distinct from OCSCC the role of EZH2 in remains unclear but may involve an interaction with the HPV-E7 oncoprotein [22, 23]. In this study, high levels of EZH2 were more often found in HPV+ OPSCC tumors. In HPV+ OPSCC cell lines, inhibition of EZH2 caused a dramatic reduction in H3K27me3 and significant depletion in cell counts. Our results suggest EZH2 has a central regulatory role in HPV+ OPSCC.

Human papillomavirus (HPV) has been identified as an additional independent risk factor for the development of HNSCC, particularly OPSCC. HPV-positive and HPV-negative OPSCCs are distinct molecular and clinical entities and have different causes, risk-factor profiles, and survival outcomes [39]. The malignant phenotype of HPV-positive cancer cells is maintained by the activity of the viral E6 and E7 genes. Viral E7 activates EZH2 in cervical cancer cells and is believed to contribute to the apoptotic resistance of HPV-transformed cells. It also believed that EZH2 expression may be necessary for the proliferation of HPV-positive tumor cells by stimulation cell cycle progression at the G_1_-S junction [23]. Our data demonstrate that the expression level of *EZH2* was significantly higher in HPV+ vs. HPV- tumors (*p* = 0.006) (Fig. 1), consistent with the results reported for HPV+ cervical cancer [23]. EZH2 expression was found to be significantly associated with Ki-67 expression as also noted in overexpression of EZH2 in OCSCC [18]. Given the association of high EZH2 levels with Ki67 seen here, we hypothesize EZH2 may have a function in cell proliferation in HPV + OPSCC.

Accumulating evidence has indicated that EZH2 serves as an essential oncogenic driving force during the initiation and progression of head neck cancers. However, the exact expression patterns of EZH2 and associated molecular mechanisms underlying head and neck tumorigenesis remains to be elucidated. Normal EZH2-mediated histone methylation process involves several key steps. One of these steps is the binding of the cofactor S-adenosyl-L-methionine (SAM) to the SAM-binding pocket in the SET-domain of EZH2 [40]. SAM, a methyl donor, is required for the catalytic reaction of HMTs, including EZH2. SAM is subsequently converted to S-adenosyl-L-homocysteine (SAH) after methyl transfer to H3K27. Finally, SAH-hydrolase catalyzes the conversion of SAH into adenosine and homocysteine. Homocysteine can then be converted back to methionine and used to generate SAM [40].

Because EZH2 is a central regulator of proliferation, migration, invasion, and stem cell properties of cancer cells, it is an appealing potential target for inhibition [29]. Numerous small-molecule EZH2 inhibitors have therefore been developed in recent years. The most commonly described EZH2 inhibitors are the SAH-hydrolase inhibitors, such as 3-Deazaneplanocin A (DZNep), and the SAM-competitive inhibitor, such as GSK343 and EPZ5687. DZNep is believed to deplete EZH2 by proteasome-mediated protein degradation, while GSK343 and EPZ00568 directly inhibit the EZH2 enzyme activity through competing with the co-factor SAM. In OCSCC, treatment with DZNep reduced EZH2 protein levels in a time- dependent and dose-dependent manner and repressed H3K27 trimethylation. Interestingly, several studies have demonstrated no significant difference in the concentration of EZH2 mRNA in the presence of DZNep and a remarkable loss of inhibitory effect of DZNep on EZH2 protein when cancer cells are treated with both DZNep and a proteosome inhibitor [30]. In our study, we have shown relatively minimal depletion of the EZH2 substrate H3K27me3 in HPV- cells, with a decrease in EZH2 but no measurable decrease in H3K27me3 in HPV+ cells. When combining data from western blots and immunofluorescence, GSK-343 appears to be a more effective EZH2 inhibitor than DZNeP and EPZ5687.

Although results of this study are promising for future investigation EZH2 inhibitors in OPSCC, we acknowledge a number of limitations. Experiments were performed in vitro on two cell lines. Further experiments with additional HPV+ and HPV- cell lines, in addition to primary cultures and in vivo models such as tumor explants would be important to further characterize the EZH2 inhibitors used in this study. The expression of EZH2 in patient tumors was performed on a relatively small sample size. Further analysis of EZH2 expression in a larger cohort of OPSCC tumors in relation to patient outcomes may provide important information about the role of this protein in OPSCC.

## Conclusions

Inhibition of EZH2 has anti-tumorigenic effects on OPSCC cells in culture that is more pronounced in HPV-positive cell lines. EZH2 is a promising epigenetic target for the treatment of OPSCC.

## Abbreviations

ddPCR: Droplet digital polymerase chain reaction
DNA: Deoxyribonucleic acid
DZNep: 3-deazaneplanocin A
EZH2: Enzyme enhancer of zeste homolog 2
H3K27me3: Trimethylated lysine 27 of histone H3
HMT: Histone methyltransferase
HNSCC: Head and neck squamous cell carcinoma
HPV: Human papillomavirus
IHC: Immunohistochemistry
OCSCC: Oral cavity squamous cell carcinoma
OPSCC: Oropharyngeal squamous cell carcinoma
RNA: Ribonucleic acid
SAH: S-adenosyl-L-homocysteine
SAM: S-adenosyl-L-methionine
